# Determinants of myocardial perfusion reserve measured from coronary sinus phase-contrast imaging during regadenoson stress CMR

**DOI:** 10.1186/1532-429X-16-S1-P219

**Published:** 2014-01-16

**Authors:** Michael A Bauml, Jaehoon Chung, Vineet Dandekar, Andrew W Ertel, Carolyn Dickens, Rosalia C Gonzalez, Afshin Farzaneh-Far

**Affiliations:** 1 Cardiology, University of Illinois Hospital & Health Sciences System, Chicago, Illinois, USA

## Background

Measurement of myocardial perfusion reserve (MPR) can potentially extend the scope of conventional myocardial perfusion imaging from detection of flow limiting epicardial stenosis to assessment of coronary microvascular function. Recent studies have suggested that MPR may improve risk stratification of patients with known or suspected CAD. MPR has traditionally been measured using PET or CMR time-intensity curves. However, these techniques are cumbersome, require radiation (for PET) and are not practical for routine clinical practice. Measurement of coronary sinus (CS) flow with phase-contrast MRI is an alternative, simple method for assessing MPR. The aim of this study was to identify the clinical determinants of MPR using this method in patients with symptoms of possible myocardial ischemia presenting for CMR stress testing.

## Methods

117 consecutive patients referred for suspected myocardial ischemia underwent a CMR stress-rest perfusion protocol. Perfusion imaging was performed at 1-minute and 15-minutes after administration of 0.4 mg of regadenoson. CS through-plane flow was measured using a phase-contrast segmented gradient echo sequence at baseline (pre) and immediately after stress perfusion (peak). MPR was calculated as peak CS flow/pre CS flow. Clinical variables were stratified by impaired MPR (<2) vs preserved MPR (≥2). Multivariable logistic regression was performed to derive the clinical predictors of impaired MPR (<2).

## Results

**Figure 1:**
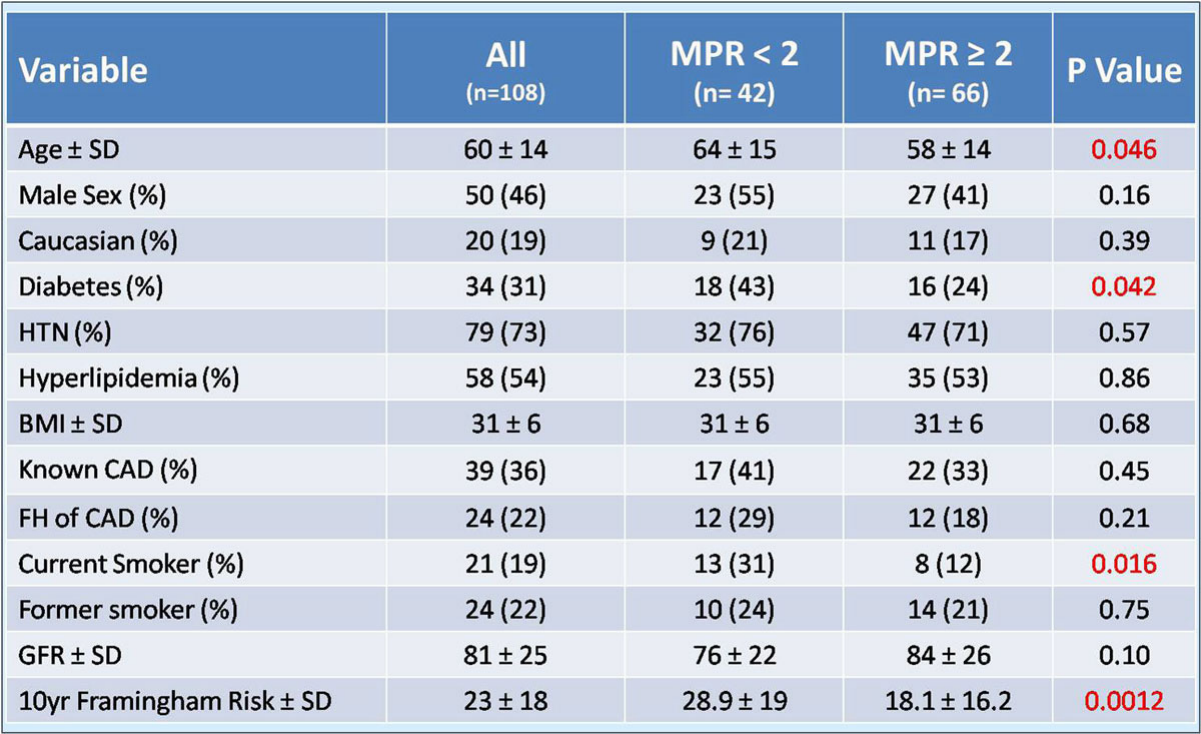


**Figure 2:**
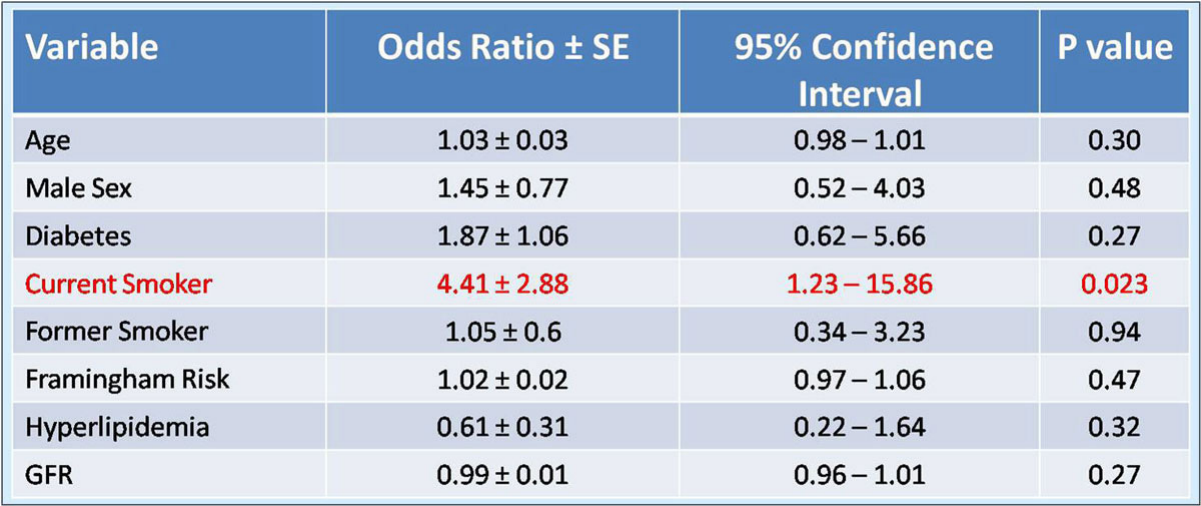


36% of the population had known CAD, 31% were diabetic and 19% were current smokers. Mean ejection fraction was 65 ± 12%. The mean 10-year Framingham risk score was 23 ± 18%. Adequate CS images were obtained in 108 patients (92%). Acquisition of CS images added approximately 2-3 minutes to overall scanning time with an additional 5 minutes required for off-line quantitative flow analysis. The flow profile obtained was typically biphasic with a first peak in early systole and a second peak during early diastole. Mean MPR in the population was 3.28 ± 0.32. Patients with MPR<2 were significantly more likely to have advanced age, diabetes, higher Framingham risk score, and history of current smoking (Figure 1). On multivariate analysis, current smoking was the only independent predictor of reduced MPR(<2) (Figure 2).

## Conclusions

MPR can be rapidly measured in the clinical setting using CS flow measurements during stress CMR. Patients with impaired MPR were significantly more likely to have advanced age, diabetes, higher Framingham risk score, and history of current smoking. On multivariate analysis, current smoking was the only independent predictor of reduced MPR. Whether this method of MPR assessment can provide independent, additive prognostic value requires further investigation.

## Funding

None.

